# Evaluation of the methanogenic potential of anaerobic digestion of agro-industrial wastes

**DOI:** 10.1016/j.heliyon.2023.e14317

**Published:** 2023-03-04

**Authors:** Rodrigo Sequeda Barros, Michel Durán Contreras, Felipe Romani Morris, Marley Vanegas Chamorro, Alberto Albis Arrieta

**Affiliations:** a Research Group KAÍ, Department of Chemical Engineering, Universidad del Atlántico, Puerto Colombia, Barranquilla Metropolitan Area-081007, Atlántico, Colombia; b Research Group Bioprocess, Department of Chemical Engineering, Universidad del Atlántico, Puerto Colombia, Barranquilla Metropolitan Area-081007, Atlántico, Colombia

**Keywords:** Anaerobic digestion, Methanogenesis, Poultry manure, Rumen, Biogas

## Abstract

Waste management technologies have become a way to generate value-added products. Anaerobic digestion (AD) allows biogas generation by treating organic wastes. In this work, the methanogenic potentials of anaerobic digestion of rumen and chicken manure, two typical agro-industrial wastes from the Colombian Caribbean region, were evaluated. On a first stage, the effect of temperature on anaerobic digestion of manure inoculated with liquid rumen was measured. Results revealed that the thermophilic digestion produces more biogas (up to 47% higher than the mesophilic digestion), but the mesophilic digestion has better biogas quality (up to 20% more methane than the thermophilic digestion). On the second experimental stage, it was assessed the effect of temperature regimen and the addition of fat-oil-grease (FOG) on cumulative biogas production, methane percentage, and physicochemical parameters. It was found that the anaerobic digestion of the rumen with FOG in mesophilic conditions had the best performance in terms of quantity and quality of biogas (2520 NL CH_4_/kg VS, CH_4_ 93%, H_2_S 1 mg/L, H_2_O 16 mg/L). Finally, rumen and manure had methane concentrations above 40% in all cases studied, after 60 days of anaerobic digestion. It was concluded that rumen and manure are good candidates for biogas generation.

## Nomenclature

AD: Anaerobic digestion
COD: Chemical oxygen demand
FOG: Fat-oil-grease
LCFAs: Long-chain fatty acids
NL: Normal liters
NTP: Normal temperature and pressure
TAN: Total ammonia nitrogen
TS: Total Solids
TSS: Total suspended solids
VS: Volatile solids
VSS: Volatile suspended solids
VFAs: Volatile fatty acids

## 1 Introduction

The marked impact of the planet's energy crisis, a product of human activity, has encouraged the development of alternative energy sources to diversify the energy matrix and reduce dependence on fossil fuels [1,2]. In this sense, studies oriented towards using clean energy generation sources and residues from industrial and agro-industrial processes to obtain their maximum utility should be evaluated to reduce the use of fossil resources and the impact of waste management [[3], [4], [5]]. Among the existing alternatives is the use of industrial and agro-industrial waste for biogas production. Biogas can be obtained by using available biomass, thus reducing greenhouse emissions: methane from biomass decomposition and CO_2_ produced by burning fossil fuels [[6], [7], [8]]. In addition, biogas versatility allows it to be used to generate heat, electricity, lighting, and mechanical power [9,10]

Among the different biogas production routes, there are thermophilic and mesophilic anaerobic digestive processes. Thermophilic digestion processes (40–60 °C) generally produce high methanogenic efficiency compared to mesophilic digestion (30–40 °C) [11]. In that sense, thermophilic digestion becomes a more economical option to operate and, in addition, allows a disinfectant effect from the elimination of the pathogens present. However, thermophilic digestion is more sensitive to changes in pH conditions, volatile fatty acids (VFA), toxic substrates, and the concentration of free ammonia in the system, which affects the stability of the operation [[12], [13], [14]]. For instance, Morya et al. [15] demonstrated that microbial digestion under anaerobic digestion could produce mixed biogas using carbon source. The free ammonia concentration in the system strongly depends on pH variations and total ammonia nitrogen (TAN). Therefore, it becomes a key inhibitor in methane production from poultry manure [16]. However, according to the results reported by Jiang et al. [17], thermophilic and mesophilic processes are inhibited at total ammonia nitrogen concentration above 2500 mg/L. Mesophilic anaerobic digestive processes using poultry manure can be carried out using contents of total solids of 1–25% [[18], [19], [20]] and organic loads in batch conditions or in single-stage systems with hydraulic retention times between 20 and 30 days [[21], [22], [23]]. Some studies report ranges of total solids (TS) from 1% to 10% obtaining good bioconversions to methane (4–6% TS) under mesophilic conditions, as reported by Webb et al. [20]. Their results suggest a multi-stage operation (hydrolysis/acidogenesis phase and methanogenic phase) with high levels of pre-adapted ammonia as an alternative to single-stage designs. Niu et al. [24] conducted a comparative study of the performance of the methanogenic activity of thermophilic and mesophilic anaerobic digestion of poultry manure, noting that the mesophilic reactor had a higher tolerance for total ammona nitrogen (TAN) than the thermophilic reactor.

On the other hand, fat-oil-grease (FOG) obtained from commercial activities has been used as a co-substrate to increase biogas production in anaerobic digestion processes. In this regard, studies presented by Tandukar et al. [25] revealed that biogas production can be increased by more than 30% when FOG is added directly to the digestion process. In co-digestion of FOG with sludge from wastewater plants, Pereira et al. [23] found an increase of 9%–27% in methane gas production. Silvestre et al. [26] investigated the co-digestion of organic fractions of municipal solid waste and FOG and found an increase in biogas produced of about 46% when oily wastes were fed at 15%. The use of FOG in the digestion process has its challenges, and they are intimately related to the potential inhibitors produced in the form of volatile fatty acids (VFAs) or long-chain fatty acids (LCFAs). However, this inhibition is not permanent [27] and can be prevented by the adaptation of anaerobic cultures and careful consideration of the ratios used to avoid excessive organic loading to the system [28].

In this regard, the microorganisms present in the rumen are a natural cellulose degradation system capable of digesting lignocellulosic biomass in specific periods [29]. In this sense, studies report high degradation efficiencies of volatile fatty acids (VFA) higher than 75% using rumen as an essential component of digestion [30,31]. Additionally, it is essential to highlight that between 23% and 27% of the total methane produced by human activities comes from ruminants due to the 10^8^-10^10^ org g^−1^ of methanogens in the rumen microbial ecosystem [32]. This indicates that rumen cultures can degrade cellulosic materials and have a high potential for conversion of VFA into biogas. Among the different studies of co-digestion processes of industrial and agro-industrial wastes with rumen are those reported by Gijzen et al. [33] and Mosoni et al. [34]. Their results revealed that the use of rumen in the anaerobic digestion processes of these wastes generates a chemical oxygen demand (COD) elimination of 90.3% with a 62% degradation efficiency of neutral detergent fiber. The rumen's potential for converting high-cellulose waste to methane has been studied using various configurations. Masoni et al. [32] studied the conversion of cellulose to methane in two stages. The first consisted of the degradation of polymeric waste into VFA and carbon dioxide. The resulting effluent was converted to methane in the second stage. The results showed a high conversion of cellulose to biogas. The polymeric sugars need to be converted into monomeric sugars for microbial consumption through enzymatic hydrolysis before transformation into VFAs. For instance, Chandrasekhar et al. [35] and Raj et al. [36] demonstrated that sugar obtained after enzymatic saccharification could be metabolized by various strains for production of variety of fuel and chemical.

Based on the above review, there is great potential in using rumen as a substrate to increase the efficiency of biogas generation from industrial and agro-industrial wastes. Additionally, anaerobic digestion has become a route to contribute to the transition from a linear to a circular economy and reduce the carbon footprint [37,38]. Therefore, conducting studies aimed at evaluating the performance of the mesophilic anaerobic digestion process of agro-industrial wastes such as rumen and poultry manure is of great importance. This kind of studies contribute to the agro-industrial sector of the Colombian Caribbean coast to increase its productivity and competitiveness by taking advantage of its waste to obtain higher value-added by-products, thus closing the circle of circular economy, and contributing to the country's energy transition. This will promote both Colombia's economic reactivation and climate change mitigation.

The main objective of this work was to study the effect of the temperature regime and the addition of FOG on the methanogenic evolution of the anaerobic digestion of poultry manure and rumen. In this way, it could support the development of the technology of biogas production in the Colombian Caribbean region and to overcome the gaps associated with the limited bibliographic availability of exploratory studies in this type of process.

## 2 Materials and methods

### 2.1 Organic waste

The study was conducted with poultry manure and rumen. The poultry manure was collected from a local broiler production farm with 40-day growing cycles. The solid sample was reduced to 400 μm to be homogenized. Additionally, most of the rice husk present in the sample was removed because of its high lignocellulosic content [[39], [40], [41]].

The rumen was obtained from a local beneficiary. The excess liquid phase (liquid rumen) was subtracted from the mass to be used as inoculum for the trials with poultry manure. The remaining (solid rumen) was used for the corresponding tests. For the co-digestion trials with solid rumen, cooking oil obtained from a local fast-food establishment was used.

### 2.2 Equipment configuration

**Fig. 1:**
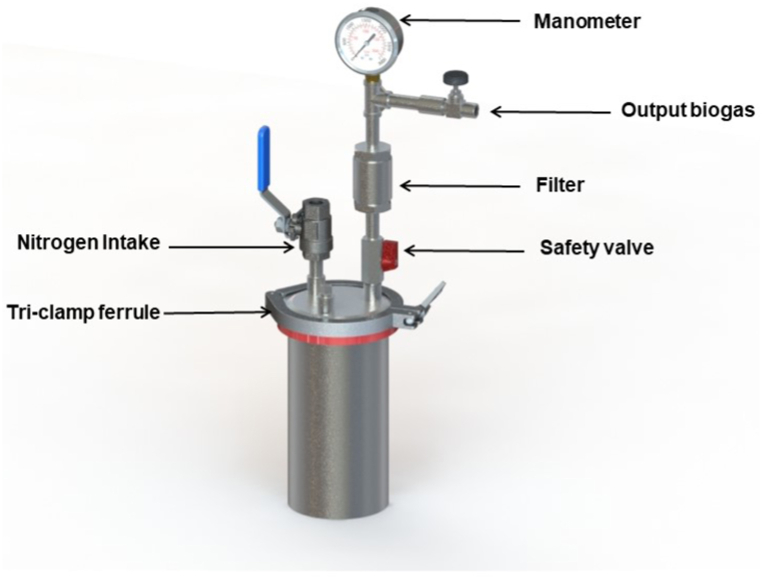
Schematic of the experimental configuration of the equipment.

For the follow-up, control, and monitoring of biogas production, 1.8 L reactors were used, consisting of hollow vessels and an end cap secured with a tri-clamp ferrule to guarantee hermeticity. The end cap has two outlets for the nitrogen intake (inert gas) and the removal of the gas produced. The equipment has a manometer for pressure reading. In addition, the equipment contains air filters and safety valves, as shown in Fig. 1. The working volume corresponds to 1.4 L.

### 2.3 Measurement of parameters and variables

Parameters such as total solids (TS), total suspended solids (TSS), volatile solids (VS), volatile suspended solids (VSS), alkalinity, and pH were measured according to standard methods for the examination of water and wastewater, Table 1.

**Table 1:** Standard methods for the examination of water and wastewater.

Parameter	Norma
Total solid	APHA/SM – 2540-B
Total suspended solids	APHA/SM – 2540-D
Volatile solids	APHA/SM – 2540-E
Alkalinity	APHA/SM – 2320-B
pH	APHA/SM – 4500 H_+_ - B

The volume of biogas generated was determined from ratios of free volume, manometric pressure, volumetric percentages of biogas components, and internal temperature [42]. This monitoring was carried out daily to analyze the progress of the production of biogas for the experimental setups. Regarding gas quality, Dräger X-AM 8000 equipment and Dräger colorimetric tubes were used to check the totality of the main species in the biogas. This allowed the comparison of the quality gas with the limits established by Colombian legislation for its use as fuel.

### 2.4 Experimental design

On a first stage, the effect of temperature (mesophilic and thermophilic conditions) on the methanogenic evolution of manure inoculated with liquid rumen was assessed using a one-factor two-level factorial design: poultry manure was inoculated with liquid rumen and anaerobic digestion was performed using either mesophilic temperature (30 °C) or thermophilic temperature (50 °C), as shown in Table 2. Data were collected for case A and case B. Each of the replicates of the cases was called a case, i.e., case A1, case A2, etc. (see supplementary material).

**Table 2:** Working conditions for each experimental design.

Designs	Cases	Descriptions
Design 1	Case A	Poultry manure with rumen fluid, thermophilic conditions (50 °C)
	Case B	Poultry manure with rumen fluid, mesophilic conditions (30 °C)
Design 2	Case C	Rumen with 0% waste FOG^a^, thermophilic conditions (50 °C)
	Case D	Rumen with 0% waste FOG, mesophilic conditions (30 °C)
	Case E	Rumen with 1% waste FOG, thermophilic conditions (50 °C)
	Case F	Rumen with 1% waste FOG, mesophilic conditions (30 °C)

a FOG = fat-oil-grease.

On a second stage, the solid rumen co-digestion with FOG, at 1% and 0% concentrations, were studied under mesophilic (30 °C) and thermophilic (50 °C) conditions, using a two-factor two-level factorial design ($2^{2}$). This design allowed investigating the effect of FOG percentage and the temperature on biogas production and the interactions between these two factors. Poultry manure and rumen were studied in different designs since a zero-inoculum composition for poultry manure varying the temperature was not within the scope of this work. The addition of fats due to the available carbon content of residual rice husk in the manure was also not studied.

The working conditions for both designs and the identification code for each run were organized as shown in Table 2. Cases A and B correspond to experiments with substrate manure, while C, D, E, and F correspond to experiments with solid rumen. All experiments were carried out by triplicate.

Analysis of variance (ANOVA) was performed using StatGraphics® Centurion XV software. The Fisher Least Significant Difference (LSD) test was used to study the statistical significance between the different assays at a 95% confidence level.

Other digestion parameters were kept constant in all experiments: total solids of 6% were chosen as a base [43,44] and the mass of raw material, water, and adjuvants to be added was determined to obtain a percentage distribution for the working volume (80%) and free space (20%). An inert atmosphere (nitrogen gas) was used to guarantee the absence of oxygen. Subsequently, the reactors were subjected to the desired temperature in thermostatic baths.

The study period was sixty days. During this time, the differential manometric pressure value was registered four times a day. Gas quality tests were performed to ensure the flow and pressure necessary for the operation of the measurement equipment. In the first work days, gas quality tests were performed according to production performance. At the end of the observation stage, final samples of the digestate were taken in the reactors to compare physicochemical parameters. These activities were carried out for each of the experiments and replicates.

## 3 Results and discussion

### 3.1 Effect of the temperature on crushed poultry manure digestion

In this section, the effect of thermophilic and mesophilic conditions on biogas production from crushed poultry manure with rumen is shown. The reactors were kept under observation for 60 days. Data of biogas and methane rate production, at the end of the experiment (day 60), are presented in Table 3. Physical-chemical parameters of the digestate, such as, COD, TSS, VSS, alkalinity, and change in pH are also shown in Table 3.

**Table 3:** Biogas production, final concentration of components presents in biogas, and changes in the parameters evaluated in cases A and B fed with rumen inoculated crushed poultry manure.

Case	Biogas Production [NL biogas/kg TS day]	Biogas composition and productivity					Change in evaluated parameters				
		CH_4_ [%v/v]	H_2_S [ppm]	H_2_O [ppm]	CH_4_ production rate [NL CH_4_/kg ST day]	Specific CH_4_ production [NL CH_4_/kg VS]	COD [mg/L]	TSS [mg/L]	VSS [mg/L]	Alkalinity [mg/L]	Initial pH – Final pH
A	0.56 ± 0.03	43 ± 1	69 ± 3	10.7 ± 0.9	0.24 ± 0.01	773 ± 43	19,200 ± 8800	6000 ± 14,000	8600 ± 11,000	4900 ± 300	7.2–6.08
B	0.39 ± 0.04	54 ± 5	>100	15 ± 0.1	0.21 ± 0.03	1376 ± 160	11,100 ± 1300	2900 ± 420	2300 ± 770	10,500 ± 15,000	7.0–6.9

*NL = normal liters standardized to Normal Temperature and Pressure (NTP); TS = total solids.

It was observed that thermophilic conditions favored biogas production with respect to the mesophilic condition by 30%. Additionally, it is observed that the effect of temperature conditions on biogas production was much greater than the dispersion of the replicates. Despite of having a higher biogas production, and a higher production rate of methane, the thermophilic conditions (Case A) showed lower specific methane production which is in accordance with the lower methane content of the biogas.

**Fig. 2:**
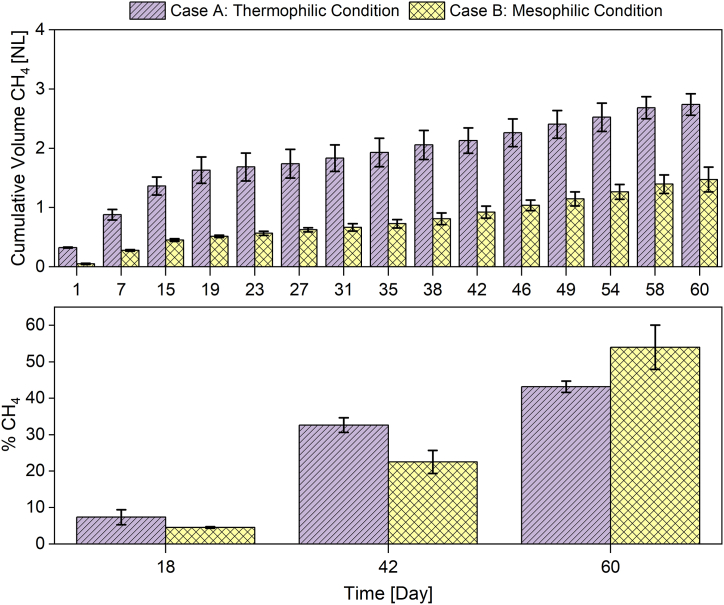
Behavior of biogas production per case: a) cumulative volume of ${CH}_{4}$, b) percentage of CH_4_.

On the other hand, the behavior of the cumulative methane production and the content of methane of the biogas for the anaerobic digestion of poultry manure over time are shown in Fig. 2. It is observed that the thermophilic (case A) and mesophilic (case B) temperatures strongly impacted the cumulative biogas production from day zero, as shown in Fig. 2a. The average cumulative biogas production of the three replicates in mesophilic conditions (case B) was 1.54 L at the end of the observation period (day 60). While in thermophilic conditions (case A), it was 2.74 L. This indicates that the thermophilic condition (case A) increased the cumulative gas production by 43.7% with respect to mesophilic conditions (case B). Therefore, production in a poultry manure system was significantly affected by temperature variations. This advantage can be favored up to a certain temperature increase. In this case, good performance at 50 °C was observed (p=0.0094<0.0500). Temperature was one of the variables that most affected the performance of microbial processes. The thermodynamic equilibrium of biochemical reactions, kinetics, stability, and others can be very sensitive to sudden changes in temperature [45].

In this sense the higher cumulative biogas production under thermophilic conditions aligns with what was reported in the literature [46]. This was because the increase in temperature favored the rate of conversion of complex organic molecules such as proteins, carbohydrates, and lipids to simpler molecules. It also favored the rate of microbial growth. In other words, the hydrolysis and acidogenic stages increased simultaneously due to the effect of temperature. The opposite case was evidenced in the mesophilic condition (case B), where a reduction in temperature retarded biogas production. This may be associated with the growth of acidifying bacteria that favor the production of soluble molecules to lactic acids and volatile fatty acids (VFAs). These acids could be a key inhibitor in these systems [47]. Despite VFAs were not measured in the experiments, initial pHs for the mesophilic experiments were higher than initial pHs for the thermophilic experiments, which suggest a higher concentration of the aforementioned acid compounds and their producer's microorganisms.

Although the thermophilic temperature (case A) caused an increase in cumulative biogas production, it had the opposite effect on the percentage of methane content. In this sense, the variations in the percentage of methane in the biogas under thermophilic (case A) and mesophilic (case B) conditions are shown in Fig. 2b. In the first 42 days, the methane percentage was higher in thermophilic conditions (case A) than concerning mesophilic conditions (case B). However, the results show an opposite behavior after the 50th day, where the methane percentage was higher in mesophilic conditions (case B) in contrast to thermophilic conditions (case B). At the end of the period (day 60), the average percentage of methane in case A and case B were 43.16% and 54%, respectively. This indicates that the mesophilic condition (case B) increased the percentage of methane contained in the biogas by 20%. Although the mesophilic condition (case B) generates a low cumulative biogas production at the end of the period (Fig. 2a), compared to the thermophilic conditions, the biogas quality was better in the mesophilic conditions (case B), as shown in Fig. 2b.

In both aspects studied (methane quality and cumulative biogas production), it was possible to note the initial increase provided to case A by the thermophilic conditions. Although it is vital to increase the efficiency of the processes, it was possible that this negatively impacted the overall performance according to the results for methane content in case A. Temperature increases the biogas and methane production rate in case A, which gives it a start-up advantage in the process. However, this advantage decreases its impact as the days go by, facilitating the start-up of the digestive process. It was possible that the production present in case A has been impetuous and has affected the chemical balance of the species, i.e., that compounds have accumulated that acidify the medium and impair the growth of the methanogenic populations present.

A pH close to neutrality must be ensured for reactor work in which the stages of anaerobic digestion are not divided. This way, acetogenic microorganisms, and methane precursors will not be affected as they have different pH ranges for optimal growth [48].

#### 3.1.1 Biogas characterization

Colombia has established gas quality specifications to be injected into the national transportation system. Likewise, it has established the permissible limits for the percentage of methane (>40%v/v) and humidity for its use in transportation networks. In this sense, it is viable to operate the system under mesophilic conditions since it guarantees that the biogas contains methane concentrations within the country's regulatory range for its use. In addition, the mesophilic condition tends to provide more stability to the system [11]. Table 3 shows each case's final concentrations of methane, hydrogen sulfide, and water vapor.

Based on Table 3, an excess of hydrogen sulfide greater than 100 ppm was observed in case B. It is common for poultry manure to generate $H_{2}S$ throughout its decomposition in an oxygen medium. Therefore, starting an anaerobic digestion process for this raw material, it is necessary to implement mitigation measures [43,49]. However, from day 44 onwards, case A showed a decreasing trend until it reached 66.5 ppm $H_{2}S$. This decrease did not occur for B (see Table 3). One of the important causes of this event was likely the operating temperature ranges. While between 20 °C and 35 °C is a suitable temperature for the growth of sulfate-reducing bacteria, at 50 °C, it is not easy for the microorganisms to carry out this metabolism unless they have been inoculated for this purpose [50]. Finally, sulforeduction in mesophilic conditions could considerably affect the consumption of acetate to obtain carbon dioxide and methane. However, no significant differences were observed in the specific methane rate production between both temperatures regimes.

#### 3.1.2 Digestate properties

Table 3 also shows the changes in the concentrations of chemical oxygen demand (COD), total suspended solids (TSS), and volatile suspended solids (VSS) during the digestion process. These values correspond to the difference between the beginning of the digestion process (day 1) and the end of the digestive process (day 60). A high degree of conversion of the organic substrate was observed due to the change in COD, TSS, and VSS in cases A, resulting in high production of organic matter. Case A3 reports an increase in the presence of solids at the end, probably due to the caking processes that disturbed the trials several times.

On the other hand, case B did not show a significant decrease in pH values at the end of the process. With this stable parameter and a slow but progressive generation, the conditions were adequate for transforming the substrate into methane in more significant quantities. The decrease in alkalinity is evidence of the destabilization of the system due to the elimination of chemical species involved in pH equilibrium. This suggests that, for this configuration, lower residence times should be fixed to avoid malfunction. Regarding chemical oxygen demand (COD) and solid concentrations (TSS and VSS), low concentration values were observed in case of B with respect to case A. This signifies a low capacity to assimilate dissolved and suspended carbon compounds present in the medium, which causes a conversion to gaseous components, i.e., methane, hydrogen sulfide, and carbon dioxide, among others. However, it is possible to establish a thermophilic regime by decreasing residence time and temperature to take advantage of these conditions' benefits [46,51].

### 3.2 Digestion of solid rumen

This section studies the effect of FOG concentrations in the rumen for thermophilic and mesophilic conditions (design 2), according to the experimental design shown in Table 2. For this case, reactor responses were recorded for 60 days. Four sets of three reactors were used, grouped in cases C, D, E, and F. Table 4 shows data of biogas and methane rate production, at the end of the experiment (day 60). Physical-chemical parameters of the digestate, such as, COD, TSS, VSS, alkalinity, and change in pH are also shown in Table 4.

**Table 4:** Biogas production, final concentration of components presents in biogas, and changes in the parameters evaluated for cases C, D, E, and F fed solid rumen.

Case	Biogas Production [NL biogas/kg TS day]	Biogas composition and productivity					Change in evaluated parameters				
		CH_4_ [%v/v]	H_2_S [ppm]	H_2_O [ppm]	CH_4_ production rate [NL CH_4_/kg ST day]	Specific CH_4_ production [NL CH_4_/kg VS]	COD [mg/L]	TSS [mg/L]	VSS [mg/L]	Alkalinity [mg/L]	Initial pH – Final pH
C	1.1 ± 0.2	85 ± 7	18 ± 9	12 ± 1	0.92 ± 0.08	917 ± 250	3200 ± 2600	−11300 ± 4400	−7500 ± 3100	−1190 ± 80	7.3–7.4
D	0.74 ± 0.06	80 ± 3	0.8 ± 0.6	10 ± 0.1	0.59 ± 0.03	2636 ± 450	7900 ± 2700	11,500 ± 940	5800 ± 1600	−490 ± 260	7.1–6.5
E	0.6 ± 0.2	66 ± 14	3.2 ± 0.7	11 ± 1	0.4 ± 0.2	434 ± 167	−6900 ± 3400	−1300 ± 2400	−3400 ± 5600	−2500 ± 840	6.9–7.7
F	0.9 ± 0.1	93 ± 4	1.0 ± 0.5	16 ± 2	0.83 ± 0.09	2520 ± 470	700 ± 1500	7100 ± 2100	6500 ± 2400	−170 ± 120	6.9–6.8

*NL = normal liters standardized to Normal Temperature and Pressure (NTP); TS = total solids.

The order from highest to lowest biogas production was: C > F > D > E. This indicates that the configuration that caused a high increase in biogas production was the rumen inoculated with 0% FOG in thermophilic conditions (case C). The results show that there was a negative effect of FOG in thermophilic condition and slightly positive in mesophilic condition, i.e., a slight increase in production was observed when the rumen was fermented with FOG in mesophilic conditions (case F, 0.8975 NL gas/kg ST day) with respect to thermophilic conditions (case E, 0.6253 NL gas/kg ST day), after 60 days of incubation. This suggests that rumen digestive processes using FOG co-substrates should be carried out under mesophilic conditions for better performance. The opposite was evident when fermenting the rumen without FOG co-substrate (case C and case D). In this scenario, the thermophilic conditions (case C) were the most appropriate as they resulted in higher biogas generation.

**Fig. 3:**
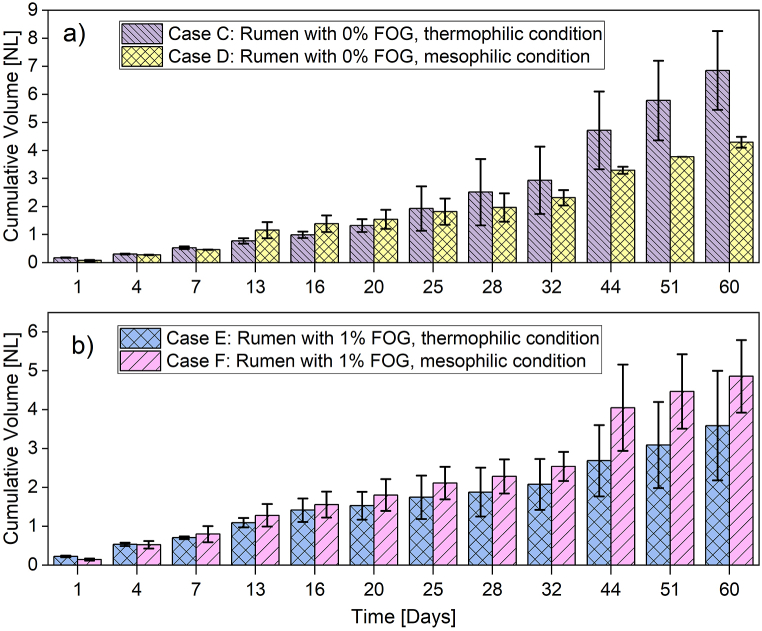
Cumulative gas volume monitoring: a) case C and D; b) case E and F.

The behavior of biogas production throughout the anaerobic digestion of rumen and anaerobic co-digestion with FOG, at mesophilic and thermophilic conditions, is shown in Fig. 3. It is observed that experiments using conditions corresponding to case C (rumen with 0% FOG at 50 °C) were the ones that dominate the generation of accumulated biogas, as shown in Fig. 3a, while the experiments corresponding to case E (rumen with 1% FOG at 50 °C) have the lowest production rate, Fig. 3b. In that sense, the ANOVA shows that biogas production was statistically significant for the combination of temperature and FOG factors (p=0.0203<0.0500). The impact of temperature (p=0.7053) and FOG (p=0.1844) individually was not statistically significant, see Table 5. Finally, although case E obtained the lowest performance in production (design 2), it was higher than the values obtained when working with crushed poultry manure with rumen (case A and case B) in the experimental design 1.

**Table 5:** Results of the Anova analysis of the studied effects.

Effect	Biogas Production [NL biogas/kg ST day]	Methane Concentration [% v/v ${CH}_{4}$]
	p-value	p-value
Temperature	0.7053	0.7053
FOG	0.1844	0.1844
Temperature and FOG	0.0203	0.0203

Additionally, it was found that cases (C, D, E, and F) comply with the definition of biogas in Colombia (minimum of 40% v/v). Case F presented the highest methane concentration, followed by case C, case D, and case E. This suggests that adding FOG in mesophilic conditions to the rumen increases methane concentration.

**Fig. 4:**
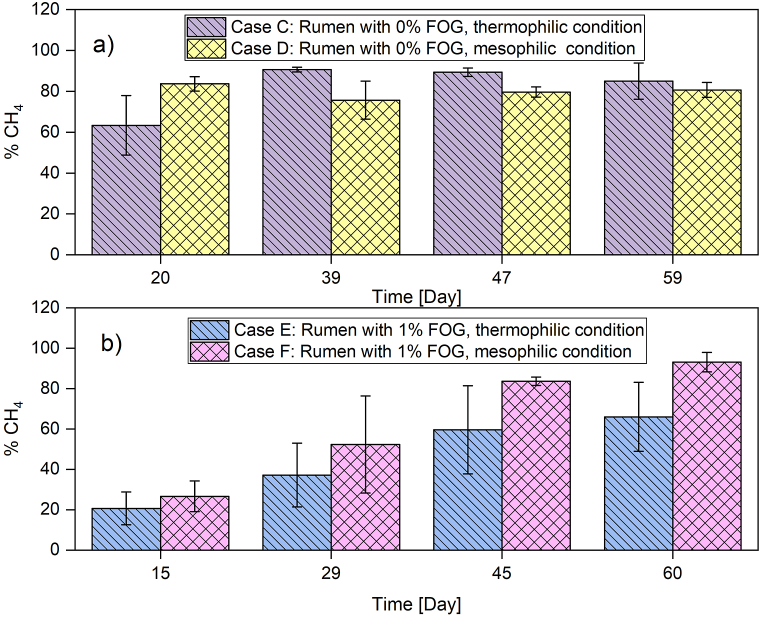
${CH}_{4}$ percentage performance in biogas: a) case C and case D; b) case E and case F.

On the other hand, Fig. 4 shows the behavior of methane percentage during the process of anaerobic digestion of rumen and its co-digestion with FOG. Although case C obtained an average value of methane content slightly lower at the end of the experiment than case F, it can be observed that the first days of these trials had higher growth than the others. According to the results in Fig. 4, values of 75% of methane were evidenced between days 25 and 30 for case C. For case F, the growth curve was lower. This could be associated with the presence of FOG in case E and case F, which dampened the increase in methane concentration until the 44th day.

Fig. 3, Fig. 4 showed that both cases C and F have a similar production of methane after 60 days of starting the experiment. However, the history of the anaerobic digestion showed differences. The anaerobic co-digestion of the studied wastes with FOG showed a beneficial effect at mesophilic conditions, but negative consequences at thermophilic conditions in terms of both biogas production and methane content. It has been reported that some methanogenic bacteria enhance biomethane production with FOG at mesophilic conditions, but thermophilic conditions promote ammonia inhibition [52].

#### 3.2.1 Biogas characterization

The values for each case for ${CH}_{4}$, $H_{2}S$, and water vapor composition are shown in Table 4. As for methane concentration, only the impact of the combination of added temperature and FOG was statistically significant (p=0.0203<0.0500). These two factors were examined separately, and the p-values were found to be non-significant for temperature (p=0.7053) and FOG (p=0.1844), see Table 5.

The hydrogen sulfide concentration in the rumen-operated case was much lower than those carried out with poultry manure. In general, thermophilic conditions promote the production of this compound, which tends to decrease as the operation time progresses, while for mesophilic conditions, it was maintained at almost imperceptible levels. All systems comply with most of the conditions stipulated in the Colombian legislation, making the rumen a valuable substrate in anaerobic digestion processes.

Table 4 shows that the presence of FOG has different effects on mesophilic and thermophilic regimes: an increase in specific methane production was found when FOG was added to rumen in mesophilic digestion, but the opposite effect was observed when digestion was carried out at higher temperatures. Differences could arise from changes on microorganisms’ populations with temperature which is a usual and highly reported behavior [[53], [54], [55], [56]]. When compared with digestion of poultry (Table 3), rumen digestion has a higher specific rate of methane production. The highest methane production was found for rumen digestion at thermophilic conditions without added FOG, followed by rumen digestion at mesophilic conditions with added FOG.

#### 3.2.2 Digestate properties

Regarding the monitoring of the physicochemical parameters, shown in Table 4, particular behaviors were found for each case. Initially, case D had an excellent performance in terms of COD and suspended solids. A slight increase in alkalinity was evidenced with respect to the other cases with a stable pH within the operating range for anaerobic digestion. Its performance in the biogas production was satisfactory when compared to the cases carried out with poultry manure. Case D can be considered a benchmark since it follows the working conditions commonly used for this type of process [57].

In this sense, it is possible to define case F as an improvement of the process, i.e., by adding FOG to the same system under mesophilic conditions of case D, better performance in the quality and quantity of biogas was achieved at the cost of a decrease in the removal rate of COD contained in the digestate. It should also be noted that adding FOG significantly improves the pH stability with respect to case D. That is, the degradation rate was reduced due to the addition of FOG. This constitutes a way of slowing down the production of short-chain acids and, therefore, the methanogenic populations. Methanogenic populations have a slower metabolism and are one of the bottlenecks of anaerobic digestion [22,48]. They can assimilate the acids generated, and the pH of the medium was not altered, which represents an inhibition for these populations.

When observing the results obtained from the experiments for case E in relation to the removal and performance of biogas, the combination of FOG present in a thermophilic medium causes a complete instability of the process with little consistency and repeatability (See Fig. 2, Fig. 3). This causes an increase in COD, suspended solids, and alkalinity, generating an abrupt increase in the pH of the medium, decreasing the possibilities of growth and reproduction in both methanogenic and acetogenic populations. This was reflected in a significant decrease in methane production under thermophilic conditions (Fig. 4b). Consequently, methane production during anaerobic digestion of the rumen with FOG was higher in mesophilic conditions (Case F) compared to thermophilic conditions (Fig. 4b). This could be explained in terms of the adaptability of the microorganisms' consortia to stressful conditions. High temperatures in thermophilic conditions and the presence of an unusual substrate (FOG) and its metabolic products (long chain fatty acids) could be stressful conditions for the aforementioned consortia. It is expected that microorganisms’ adaptation will be faster when only one stressful condition is present. Therefore, microorganisms in rumen, which are adapted to mesophilic conditions, should adapt faster to anaerobic co-digestion with FOG at mild temperatures. This led to lower methane production at high temperatures and 1% FOG, as reported in this work.

Finally, the physicochemical parameters measured for case C show similar characteristics in case E. This may be associated with thermophilic conditions. In addition, its biogas performance was favorable, unlike case E. It can be deduced that the temperature helps the degradation of the larger particles present in the digestate, supported by the increase in suspended solids [58], which explains the net increase in this parameter. It is also known that the temperatures of thermophilic conditions are suitable for the growth of microbiological populations since they increase the basicity from the presence of ammonia [48,59]. This was evidenced by comparing the differences in mesophilic and thermophilic cases alkalinity.

Concerning the above results, it was found that in each of the aspects evaluated, case E was the least favorable configuration. Case F, despite having a lower average net biogas production in its cases with respect to C, achieved high methane percentages and more excellent stability in terms of reduction of COD, suspended solids, and a more stable pH and alkalinity (Table 4). This makes it the best option with respect to the options evaluated for solid rumen.

## 4 Conclusions

This work evaluated the methanogenic potential of anaerobic digestion of rumen and poultry manure, two typical agro-industrial wastes from the Colombian Caribbean region. Two experimental designs were proposed to study system variables' effect on cumulative biogas production, methane percentage, total solids, pH, and alkalinity. The main conclusions are listed below.

Regarding the anaerobic bio-digestion of poultry manure, it was found that the thermophilic condition increased the accumulated biogas production by 47% with respect to the mesophilic condition. However, the mesophilic condition presented values in the percentage of methane in the biogas that was 20% higher in contrast to the thermophilic condition. In other words, the thermophilic condition favored quantity, while the mesophilic condition favored quality. Therefore, anaerobic digestion of liquid rumen inoculated poultry manure should be carried out under mesophilic conditions.

In the case of the digestion of solid rumen, the addition of FOG to the solid rumen under mesophilic conditions was the most favorable since it provided the best performance in terms of quality, complying with the percentage methane established by Colombian regulations.

## Author contribution statement

Marley Cecilia Vanegas, Alberto Albis Arrieta: Performed the experiments; Contributed reagents, materials, analysis tools or data; Wrote the paper.

Rodrigo Sequeda Barros, Michel Durán Contreras, Felipe Romani Morris: Conceived and designed the experiments; Analyzed and interpreted the data.

## Funding statement

This work was supported by the Chemical Engineering Program at the Universidad del Atlántico

## Data availability statement

Data included in article/supp. Material/referenced in article.

## Declaration of interest's statement

The authors declare that they have no conflict of interest.
