# Supplementary material for: Evaluation of the methanogenic potential of anaerobic digestion of agro-industrial wastes
Source: Heliyon. 2023 Mar 4;9(3):e14317. doi: 10.1016/j.heliyon.2023.e14317 (PMC10018565; doi:10.1016/j.heliyon.2023.e14317)
Supplement: Multimedia component 1 [file mmc1.docx]

**Supplementary material of the article: Evaluation of the methanogenic potential of anaerobic digestion of agro-industrial wastes.**

**Equations**

Below are presented a brief description of the procedures and equations for calculations of physicochemical parameters:

Total solids (TS) were measured according to APHA/SM – 2540-B. A known volume of a representative sample is evaporated in a suitable pan, and TS was calculated as:

$$TS = \frac{\left( A-B \right)*1000}{V}$$

where *TS* are the total solids in mg/L, *A* is the weight of the pan and the dry residue (mg), *B* is the weight of the pan (mg), and *V* is the volume of the sample in mL.

Total suspended solids (TSS) were measured according to APHA/SM – 2540-D. Briefly, a known volume of a representative sample is filtered, and the retained residue is dried. TSS is calculated as:

$$TSS = \frac{\left( C-D \right)*1000}{V}$$

where TSS are the total suspended solids expressed in mg/L, *C* is the weight of the filter and the residue (mg), *D* is the weight of the filter (mg), and *V*, the volume of the sample in mL.

Volatile solids (VS) were measured using APHA/SM – 2540-E. The residue of TS was calcinated at 500 °C and VS calculated as:

$$VS = \frac{\left( E-F \right)*1000}{V}$$

where VS is expressed in mg/L, *E* and *F* are the mass (mg) of the residue and pan before and after the calcination, respectively. *V* is the volume of the sample in mL.

Volatile suspended solids (VSS), in mg/L, is measured from the calcination of the residue of TSS and calculated as:

$$VSS = \frac{\left( G-H \right)*1000}{V}$$

where *G* and *H* are the mass (mg) of the residue and pan before and after the calcination, respectively. *V* is the volume of the sample in mL.

Finally, the alkalinity was measured according to APHA/SM – 2320-B. This is a volumetric method, and the alkalinity was calculated as:

$$Alkalinity= \frac{I*N*50000}{V}$$

where the alkalinity is expressed in mg CaCO_3_/mL, *I* and *N* are the volume of the acid used in the titration (mL) and its normality, respectively. *V* is the volume of the sample in mL.

**Experimental data**

**Table S1**. Biogas production in cases A and B fed with rumen inoculated crushed poultry manure.

| **Case** | **Replica** | **Production**  **[NL biogas/ kg TS day]^*^** |
| --- | --- | --- |
| A | 1 | 0.5881 |
|  | 2 | 0.5823 |
|  | 3 | 0.5190 |
| B | 1 | 0.4185 |
|  | 2 | 0.3293 |
|  | 3 | 0.4189 |

^*^NL = normal liters standardized to Normal Temperature and Pressure (NTP); TS= total solids

**Table S2.** Final concentration of components presents in biogas for poultry manure cases.

| **Case** | **Replica** | **Methane**  **[% v/v]** | **Hydrogen sulfide [ppm]** | **Water vapor concentration [ppm]** | **Methane Production  [NL** $\mathbf{CH}_{\mathbf{4}}$**/ kg ST day]** | **Methane Production**  **[mL** $\mathbf{CH}_{\mathbf{4}}$**/ kg VS]** |
| --- | --- | --- | --- | --- | --- | --- |
| **A** | 1 | 43.5 | 67.5 | 10.0 | 0.256 | 786890.77 |
|  | 2 | 41.5 | 73.0 | 10.0 | 0.242 | 818238.39 |
|  | 3 | 44.5 | 66.5 | 12.0 | 0.231 | 713704.85 |
| **B** | 1 | 61.0 | >100 | 15.0 | 0.255 | 1559022.50 |
|  | 2 | 50.0 | >100 | 15.0 | 0.165 | 1169133.75 |
|  | 3 | 51.0 | >100 | 15.0 | 0.214 | 1400484.81 |

**Table S3**. Change in the parameters evaluated in cases A and B fed with crushed poultry manure inoculated with rumen liquid.

| Case | Replica | COD  [$\mathbf{mg L}^{\mathbf{-1}}$] | TSS  [$\mathbf{mg L}^{\mathbf{-1}}$] | VSS  [$\mathbf{mg L}^{\mathbf{-1}}$] | Alkalinity [$\mathbf{mg L}^{\mathbf{-1}}$] | pH |
| --- | --- | --- | --- | --- | --- | --- |
| A | 1 | 27425 | 23693 | 22548 | 4853 | 7.16 - 6.11 |
|  | 2 | 23210 | 6000 | 8916 | 4570 | 7.23 - 6.15 |
|  | 3 | 6970 | -12000 | -5500 | 5321 | 7.20 - 5.98 |
| B | 1 | 11667 | 2750 | 2750 | 32069 | 7.05 - 6.65 |
|  | 2 | 12333 | 3500 | 3000 | -1354 | 6.95 - 7.08 |
|  | 3 | 9367 | 2500 | 1250 | 985 | 6.87 - 6.98 |

**Table S4**. Cumulative biogas volume of cases C, D, E, and F fed solid rumen.

| **Case** | **Replica** | **Production**  **[NL biogas/ kg ST day]^*^** |
| --- | --- | --- |
| **C** | 1 | 1.012 |
|  | 2 | 1.354 |
|  | 3 | 0.930 |
| **D** | 1 | 0.822 |
|  | 2 | 0.694 |
|  | 3 | 0.706 |
| **E** | 1 | 0.891 |
|  | 2 | 0.576 |
|  | 3 | 0.408 |
| **F** | 1 | 0.894 |
|  | 2 | 0.734 |
|  | 3 | 1.063 |

^*^NL = normal liters standardized to Normal Temperature and Pressure (NTP); TS= total solids

**Table S5.** Final concentration of components presents in biogas for solid rumen cases.

| **Case** | **Replica** | **Methane**  **[% v/v]** | **Hydrogen sulfide [ppm]** | **Water vapor concentration [ppm]** | **Methane production [NL** $\mathbf{CH}_{\mathbf{4}}$**/ kg ST day]** | **Methane production [mL** $\mathbf{CH}_{\mathbf{4}}$**/ kg VS]** |
| --- | --- | --- | --- | --- | --- | --- |
| C | 1 | 92.0 | 29.00 | 13.0 | 0.931 | 828931.13 |
|  | 2 | 75.0 | 6.00 | 12.0 | 1.016 | 1261546.78 |
|  | 3 | 88.0 | 20.20 | 10.0 | 0.819 | 659694.56 |
| D | 1 | 76.3 | ≈0.00 | 10.0 | 0.627 | 2411577.46 |
|  | 2 | 84.0 | 1.30 | 10.0 | 0.583 | 3266798.68 |
|  | 3 | 79.6 | 1.20 | 10.0 | 0.562 | 2229010.85 |
| E | 1 | 82.0 | 2.60 | 11.0 | 0.731 | 640906.11 |
|  | 2 | 68.0 | 4.20 | 13.0 | 0.392 | 229980.29 |
|  | 3 | 48.0 | 2.90 | 10.0 | 0.196 | 431833.24 |
| F | 1 | 95.7 | 1.76 | 13.0 | 0.856 | 2780892.09 |
|  | 2 | 96.2 | 0.70 | 18.0 | 0.706 | 1864102.93 |
|  | 3 | 87.6 | 0.70 | 16.0 | 0.932 | 2916070.65 |

**Table S6.** Parameters evaluated in cases C, D, E, and F fed solid rumen.

| Case | Replica | COD  [$\mathbf{mg L}^{\mathbf{-1}}$] | TSS  [$\mathbf{mg L}^{\mathbf{-1}}$] | VSS  [$\mathbf{mg L}^{\mathbf{-1}}$] | Alkalinity [$\mathbf{mg L}^{\mathbf{-1}}$] | pH |
| --- | --- | --- | --- | --- | --- | --- |
| C | 1 | 6533 | -9000 | -6600 | -1086 | 7.44 - 7.60 |
|  | 2 | 2933 | -7500 | -4250 | -1223 | 7.39 - 7.34 |
|  | 3 | 167 | -17500 | -11750 | -1267 | 7.13 - 7.30 |
| D | 1 | 9367 | 12500 | 4250 | -845 | 6.86 - 6.51 |
|  | 2 | 10301 | 11750 | 8000 | -219 | 7.18 - 6.51 |
|  | 3 | 4000 | 10250 | 5000 | -415 | 7.25 - 6.40 |
| E | 1 | -5270 | 0 | -250 | -1365 | 6.78 - 7.53 |
|  | 2 | -11640 | -4750 | -11250 | -2805 | 6.95 - 7.23 |
|  | 3 | -3800 | 750 | 1250 | -3363 | 6.92 - 8.38 |
| F | 1 | -933 | 9750 | 9750 | -196 | 7.45 - 6.23 |
|  | 2 | 400 | 4,750 | 4,000 | -301 | 6.72 - 6.91 |
|  | 3 | 2,666 | 6,750 | 5,750 | -4 | 6.61 - 7.16 |
